# Discovery of the interfamily grafting capacity of *Petunia*, a floricultural species

**DOI:** 10.1093/hr/uhab056

**Published:** 2022-01-20

**Authors:** Ken-ichi Kurotani, Chaokun Huang, Koji Okayasu, Takamasa Suzuki, Yasunori Ichihashi, Ken Shirasu, Tetsuya Higashiyama, Masaki Niwa, Michitaka Notaguchi

**Affiliations:** 1 Bioscience and Biotechnology Center, Nagoya University, Furo-cho, Chikusa-ku, Nagoya 464-8601, Japan; 2 Graduate School of Bioagricultural Sciences, Nagoya University, Furo-cho, Chikusa-ku, Nagoya 464-8601, Japan; 3 College of Bioscience and Biotechnology, Chubu University, Matsumoto-cho, Kasugai 487-8501, Japan; 4 Center for Sustainable Resource Science, RIKEN, Tsurumi, Yokohama, Kanagawa 230-0045, Japan; 5 RIKEN BioResource Research Center, Tsukuba, Ibaraki 305-0074, Japan; 6 Graduate School of Science, University of Tokyo, Yayoi, Bunkyo-ku, Tokyo 113-8657, Japan; 7 Institute of Transformative Bio-Molecules, Nagoya University, Furo-cho, Chikusa-ku, Nagoya 464-8601, Japan; 8 Graduate School of Science, Nagoya University, Furo-cho, Chikusa-ku, Nagoya 464-8601, Japan; 9 GRA&GREEN Inc., Incubation Facility, Nagoya University, Furo-cho, Chikusa-ku, Nagoya 464-8601, Japan

## Abstract

In grafting, an agricultural technique for propagating flower species and fruit trees, two plants are combined to exploit their beneficial characteristics, such as rootstock disease tolerance and vigor. Grafting incompatibility has been observed, however, between distantly related plant combinations, which limits the availability of plant resources. A high grafting capacity has been found in *Nicotiana*, belonging to Solanaceae, but not in *Ipomoea nil*, a Convolvulaceae species. Here, we found that *Petunia hybrida*, another solanaceous species, has similar ability of interfamily grafting, which indicates that interfamily grafting capability in Solanaceae is not limited to the genus *Nicotiana*. RNA sequencing-based comparative time-series transcriptomic analyses of *Nicotiana benthamiana*, *I. nil*, and *P. hybrida* revealed that *N. benthamiana* and *P. hybrida* share a common gene expression pattern, with continued elevated expression of the *β-1,4-glucanase* subclade gene *GH9B3* observed after interfamily grafting. During self-grafting, *GH9B3* expression in each species was similarly elevated, thus suggesting that solanaceous plants have altered regulatory mechanisms for *GH9B3* gene expression that allow tissue fusion even with other species. Finally, we tested the effect of the β-1,4-glucanase inhibitor D-glucono-1,5-lactone, using glucose as a control, on the interfamily grafting usability of *P. hybrida* with Arabidopsis rootstock. Strong inhibition of graft establishment was observed only with D-glucono-1,5-lactone, thus suggesting the important role of *GH9B3* in *P. hybrida* grafting. The newly discovered grafting compatibility of *Petunia* with different families enhances the propagation techniques and the production of flower plants.

## Introduction

The garden petunia*, Petunia hybrida* (Solanaceae: Solanales), is a floricultural plant first introduced to Europe from South America in the early 19th century [1]. The native region of this species is considered to be the foothills of the Andes, with its current distribution ranging from Argentina to Uruguay and southern Brazil [1, 2]. *P. hybrida* is derived from its close relative *P. axillaris* and one or more species within the *P. integrifolia* complex [1, 3]. Other floricultural species in Solanales include jasmine tobacco (*Nicotiana alata*) and morning glory (*Ipomoea nil*). *Petunia* has remained one of the most popular genera in the U.S., Europe, and Asia, with many varieties still being produced. *Petunia* noted for its suitability as a model for plant research at the same period that Arabidopsis was recognized as a model plant [4]. The plant is easy to grow and has a relatively short life cycle from seed to seed, approximately 3 to 4 months. *Petunia* can be easily asexually propagated from cuttings, calli, or protoplasts and is readily transformed [5]. Genomic analysis of *Petunia* started prior to 1980, and genetic maps based on mutants have been generated for seven pairs of chromosomes [6]. Analysis remains incomplete, however, in large part because many varieties of *Petunia,* including *P. hybrida*, are hybrid amphidiploids [7].

*Petunia* can be easily grafted [8]. Grafting, an agricultural technique used since ancient times to cultivate plants, combines the advantages of two varieties [9]. Grafting that combines a productive scion with rootstock adapted to soil characteristics is used for the production of various agricultural crops but has significant limitations [9, 10], i.e. grafting is usually only possible between plants that are genetically closely related. When two distantly related plants, such as those from different families, are grafted to each other, the tissues at the junction do not adhere, and the scion dies and drops out; such “interfamily grafting” has thus been considered almost hard to achieve [11, 12, 13]. When *Nicotiana benthamiana*, a model plant in Solanaceae, is grafted onto plants from other families, however, the connection sites are firmly attached, which indicates that the scion can survive for long periods of time [14]. A time-series transcriptome analysis of *N. benthamiana* and Arabidopsis grafting has revealed that the *GH9B3* gene, which encodes one of the subclades of β-1,4-glucanase, is expressed during grafting and promotes cell wall adhesion [14].

Grafting is an artificial union between plant tissues, but similar phenomena exist in nature as well. Parasitic plants deprive their hosts of water and nutrients by developing specialized organs called haustoria that adhere to the stem and roots of the host plants and bind their own vascular system [15]. The model parasitic plant *Phtheirospermum japonicum* (Orobanchaceae: Lamiales) can naturally parasitize distantly related plants belonging to different families [16, 17]. This hemiparasite, as well as members of the holoparasitic genus *Cuscuta*, can also be artificially grafted onto other plants from different families [18, 19]. In *Phtheirospermum japonicum*, expression of *GH9B3* is not only enhanced during interfamily grafting but is also upregulated during parasitism. Transient suppression of *GH9B3* expression prevents the haustorium, formed during parasitism, from adhering to the host, thus suggesting this gene is also essential for tissue adhesion during parasitism.

In this study, we found that *Petunia* is also capable of establishing grafting with diverse plant species. Through a comparative time-series transcriptome analysis, we identified a group of genes commonly upregulated in two Solanaceae plants, which are graft compatible with different families. These identified genes included *GH9B3*, which has been proposed to play an important role in cell wall adhesion during grafting.

## Results

### Petunia has the capability for interfamily grafting

**Figure 1:**
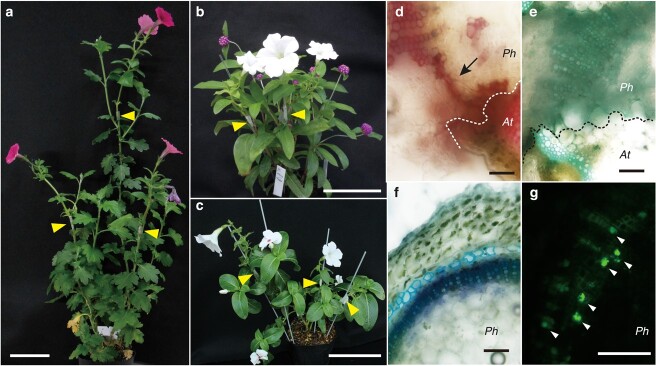
Grafts of *Petunia hybrida* with species from different families **a**–**c** Grafting of *P. hybrida* as the scion with stems of *Chrysanthemum morifolium* at 33 days after grafting (DAG) (a), *Gomphrena globosa* at 75 DAG (b), and *Catharanthus roseus* at 58 DAG (c). Arrowheads indicate grafting points. **d**–**g** Hand-cut cross-sections of *P. hybrida*/Arabidopsis graft junction regions (d, e) and the stem of *P. hybrida* scion (f, g). Dashed lines indicate the graft junction surfaces. **d** Phloroglucinol-stained transverse section indicating local xylem bridge from the scion to the stock (arrow). **e**, **f** Transverse sections after toluidine blue treatment to the cut end of Arabidopsis rootstock stem at 14 DAG. Toluidine blue dye was detected in the *P. hybrida* scion across the graft junction (e) and the stem of *P. hybrida* scion (f). **g** Transverse section of the *P. hybrida* scion stem after carboxyfluorescein (CF) diacetate treatment to the leaves of an Arabidopsis stock at 14 DAG. *Ph*, *P. hybrida*, *At*, Arabidopsis. Scale bars, 10 cm (a–c) and 100 μm (d–g).

In a previous study, we demonstrated that plants in the genus *Nicotiana* (Solanaceae), including *N. benthamiana*, can establish grafting with distantly related plants from different families (interfamily grafting) [14]. We also found that such heterofamilial grafting does not occur in *I. nil*, which is a member of the same order (Solanales) but belongs to a different family, Convolvulaceae. We hypothesized that this interfamily grafting capability was a derived trait in *Nicotiana* or a closely related genus in Solanaceae. In the present study, we therefore tested whether *P. hybrida,* another species in Solanaceae, is capable of interfamily grafting. We found that a *Petunia* scion was able to fuse with a *Chrysanthemum morifolium* (Asteraceae: Brassicaceae) rootstock in a graft union, which was followed by growth and flowering for over a month (Fig. 1a). The longest observed case was able to survive and grow for 5 months after grafting. We established grafting of *Petunia* with 17 species of plants in seven families (Fig. 1a–c, Table 1). We grafted *P. hybrida* onto Arabidopsis rootstock and tested for apoplasmic and symplasmic transport. Phloroglucinol staining of the transverse section across the graft junction revealed local xylem bridges between the stock and scion (Fig. 1d). We also grafted *P. hybrida* onto Arabidopsis rootstock, cut the Arabidopsis stem at 14 days after grafting (DAG), and allowed the stem to absorb toluidine blue from the cut end. After 24 h, transverse hand sections of the graft boundary and the scion *P. hybrida* were prepared and examined under an optical microscope. Toluidine blue dye was detected in the grafted area, not only in Arabidopsis, but also in *P. hybrida* tissue of the scion. Blue staining was also observed in the transverse section away from the grafted surface, mainly in the xylem, thus suggesting that apoplasmic transport was established (Fig. 1e, f). In addition, establishment of the symplasm between *P. hybrida* and Arabidopsis was confirmed by using carboxyfluorescein diacetate, a symplasmic tracer dye (Fig. 1g).

**Table 1:** Grafting experiments performed with *Petunia hybrida*

Scion		Stock		Trial	Success	Note
Family	Species	Family	Species			
Solanaceae	*P. hybrida*	Buxaceae	*Pachysandra terminalis*	4	1	
Solanaceae	*P. hybrida*	Brassicaceae	*Brassica oleracea var. capitata*	22	13	Sum of data from 2 experiments
Solanaceae	*P. hybrida*	Brassicaceae	*Brassica oleracea var. italica*	28	13	Sum of data from 6 experiments
Solanaceae	*P. hybrida*	Brassicaceae	*Arabidopsis thaliana*	6	6	
Solanaceae	*P. hybrida*	Brassicaceae	*Matthiola incana*	6	3	Sum of data from 2 experiments
Solanaceae	*P. hybrida*	Amaranthaceae	*Gomphrena globosa*	41	19	Sum of data from 10 experiments
Solanaceae	*P. hybrida*	Amaranthaceae	*Celosia argentea*	4	1	Sum of data from 2 experiments
Solanaceae	*P. hybrida*	Apocynaceae	*Vinca major*	2	1	
Solanaceae	*P. hybrida*	Apocynaceae	*Catharanthus roseus*	2	1	
Solanaceae	*P. hybrida*	Asteraceae	*Chrysanthemum molifolium*	60	30	Sum of data from 8 experiments
Solanaceae	*P. hybrida*	Asteraceae	*Stevia rebaudiana*	4	3	
Solanaceae	*P. hybrida*	Asteraceae	*Senecios cineraria*	5	5	
Solanaceae	*P. hybrida*	Asteraceae	*Callistephus chinensis*	3	2	
Solanaceae	*P. hybrida*	Asteraceae	*Cosmos sulphureus*	5	0	Sum of data from 2 experiments
Solanaceae	*P. hybrida*	Asteraceae	*Argyranthemum frutescens*	16	7	Sum of data from 3 experiments
Solanaceae	*P. hybrida*	Lamiaceae	*Coleus*	14	6	Sum of data from 2 experiments
Solanaceae	*P. hybrida*	Acantheae	*Crossandra infundibuliformis*	8	2	Sum of data from 2 experiments
Solanaceae	*P. hybrida*	Solanaceae	*Petunia x hybrida*	51	38	Sum of data from 6 experiments

### Transcriptome analyses of grafting and wounding in *petunia*

**Figure 2:**
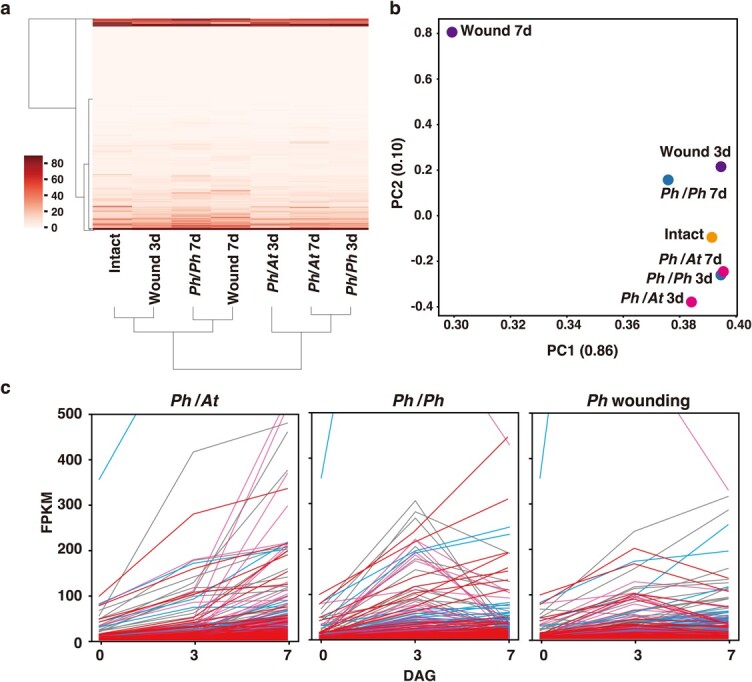
Transcriptomic analysis of *Petunia hybrida* subjected to interfamily grafting, self-grafting, and wounding Transcriptomic analysis performed using RNA samples of *P. hybrida* grafts and wounding sites. **a** Results of hierarchical clustering based on Euclidean distances and Ward’s minimum variances calculated from the ratio of RNA-seq data from two time points, 3 and 7 DAG, of *P. hybrida*/Arabidopsis grafting, *P. hybrida*/*P. hybrida* grafting, and *P. hybrida* separated upper regions vs. intact plants. **b** Principal component (PC) plot generated from the obtained expression profiles. Parentheses represent the contribution rate of each component. **c** Identification of genes upregulated during *P. hybrida*/Arabidopsis grafting. Genes whose expressions at 3 DAG were more than twice those of intact plants and whose expressions at 7 DAG exceeded those at 3 DAG were extracted, and their expression profiles were compared among *P. hybrida*/Arabidopsis grafting, *P. hybrida*/*P. hybrida* grafting, and separated upper regions of *P. hybrida*. Colors indicate comparative expression patterns of different genes as follows: red, a similar pattern in interfamily grafts and self-grafts, but reduced expression in the wounding treatment at 7 DAG; magenta, increased expression only in interfamily grafts; light blue, a similar pattern among interfamily grafts, self-grafts, and wounded samples; and gray, other patterns. *Ph*, *P. hybrida*; *At*, Arabidopsis.

Because *Petunia* exhibits interfamily grafting capabilities, we performed a transcriptomic analysis. In our previous observations, adhesion of grafted tissues occurred around 3 DAG of interfamily grafting and the graft union was stabilized by 7 DAG [14, 18]. Therefore for the time series, RNA was extracted from plant samples of intact, 3 and 7 DAG, and an RNA sequencing (RNA-seq) analysis was performed. We also compared grafting with wound repair because of the proposed commonality between grafting of the same species (self-grafting) and the mechanism of wound repair of injured plants [20, 21]. For the *Petunia* cultivar, we used *P. hybrida*, which is a hybrid of *P. axillaris* and *P. integrifolia* and has an amphidiploid genome. We mapped the generated sequence reads against the merged cDNA sequences of *P. axillaris* and *P. inflata*, a close relative of *P. integrifolia*, which served as reference data (Supplementary Table S1). Because the DNA sequences of many genes were identical between *P. axillaris* and *P. inflata*, we first solely analyzed the expressions of transcripts mapped to *P. axillaris* and performed hierarchical clustering and principal component analyses (PCA) (Fig. 2a, b). In the hierarchical clustering dendrogram, the gene expression profile of intact plants was distinct from those of either type of grafted plants, and the wounded plants are in between them. At 7 DAG, however, the expression profile of self-grafted plants was closer to that of intact plants, whereas the expression profiles of interfamily grafted plants were even more distant (Fig. 2a). The results of PCA were similar, but the gene expression profile of wounded plants at 7 DAG was quite different from that of all other analyzed samples at each time point (Fig. 2b).

We then searched for upregulated patterns in *P. hybrida*. Our gene extraction criteria were as follows: we selected genes whose expressions at 3 DAG in interfamily grafts of *P. hybrida* were more than twice as high as in intact plants and whose expression levels at 7 DAG exceeded those at 3 DAG. As a result, 668 genes were extracted. We plotted the expression patterns of these 668 genes in *P. hybrida* subjected to three different treatments: interfamily grafting, self-grafting, and wounding (Fig. 2c). On the basis of their expression patterns under the three treatments, the genes were divided into four classes: class 1 (128 genes), corresponding to genes whose expressions were similar in interfamily-grafted vs. self-grafted plants but reduced in wounded plants at 7 DAG; class 2 (173 genes), genes whose expressions continued to increase only in interfamily grafted plants; class 3 (166 genes), genes whose expressions were similar among interfamily grafted, self- grafted, and wounded plants; and class 4 (201 genes), all others. Genes belonging to each class are listed in Supplementary Table S2. We also performed a GO analysis of genes in the three classes and observed several trends (Supplementary Tables S3–S4). Genes assigned to class 1, potentially the most relevant to graft establishment, included many genes encoding protein kinases (Supplementary Table S2). Genes categorized into class 2 might be specific to typical events in interfamily grafting, where stressful conditions are prolonged before graft healing, rather than self-grafting. This class included genes related to stress response, such as peroxidase superfamily proteins (Supplementary Table S2). Class 3, with genes possibly related to wounding itself, included genes related to cell proliferation and meristem maintenance (Supplementary Table S2).

### Transcriptomic analysis of *P. hybrida* revealed the expression status of conventional graft-related genes

**Figure 3:**
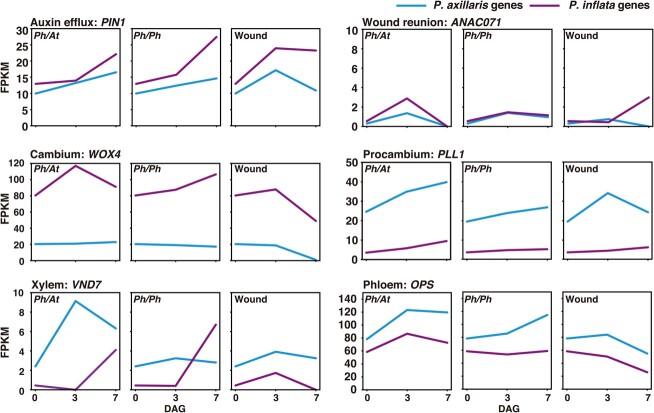
Expression patterns of genes related to conventional grafting Expression patterns of genes involved in grafting establishment in *Petunia hybrida*/Arabidopsis interfamily grafts, *P. hybrida* self-grafts, and wounded samples of *P. hybrida*. DAG, days after grafting/wounding; *Ph*, *P. hybrida*; *At*, Arabidopsis.

Expression patterns of various genes proposed to be involved in plant graft establishment [21, 22, 23] were examined in *P. hybrida* subjected to interfamily grafting, self-grafting, and wounding treatments (Fig. 3). In particular, we examined expression patterns of the following genes: *PIN1*, which is involved in auxin efflux to induce cell proliferation during grafting; *ANAC071*, a NAC domain transcription factor gene involved in wound repair; *WOX4*, which maintains cambium activity; *PLL1*, a gene involved in maintenance of procambium; *VND7*, which induces protoxylem vessel elements; and *OPS*, a regulator of phloem differentiation. Genome sequences of *P. axillaris* and *P. inflata* are highly similar; in the case of the above six genes, we accordingly found copies derived from each species in *P. hybrida*. The expression patterns of genes from *P. axillaris* and *P. inflata* were generally similar. IDs of genes that were examined for expression in *P. hybrida* are listed in Supplementary Table S6. In *P. hybrida,* these genes tended to be expressed in similar level between interfamily- and self-grafting or more highly in interfamily grafted plants than in self-grafted plants, which is consistent with previous results for *N. benthamiana* [14]. Expression patterns observed under wounding treatment were not very consistent with those uncovered during interfamily- or self-grafting. *PIN1* and *PLL1* expressions continued to increase after grafting in *P. hybrida.* In the wounding treatment, however, these genes were upregulated at 3 days after wounding and then downregulated. *ANAC071* was upregulated at 3 DAG during grafting and then was downregulated. *WOX4* and *OPS* expressions continued to rise at 7 DAG in the self-grafting treatment but were reduced after 3 DAG during interfamily grafting. During interfamily- and self-grafting, expression of the *P. axillaris* copy of the *VND7* gene was elevated at 3 DAG and returned to the same level as intact plants at 7 DAG; in contrast, the *P. inflata*-type gene first exhibited elevated expression at 7 DAG, with little increase at 3 DAG. These gene expression patterns are similar to those observed in our previous *Nicotiana* grafting experiments.

### Comparison of gene expression profiles during grafting of three Solanales species

**Figure 4:**
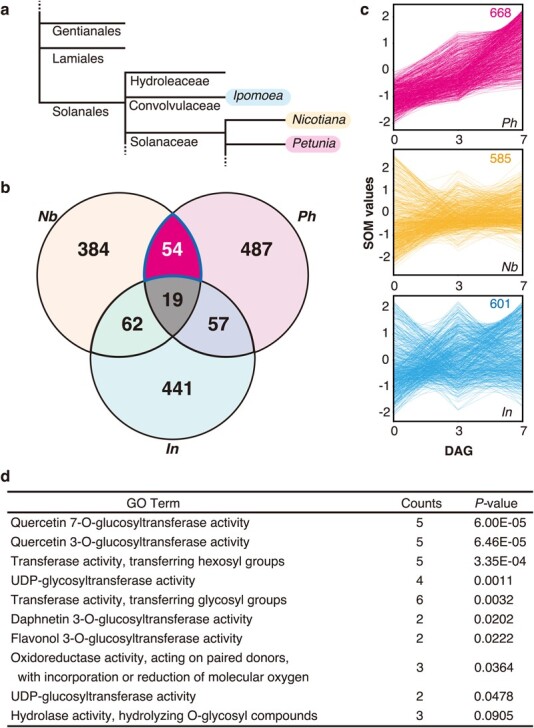
Comparison of gene expression profiles during grafting of three Solanales species **a** Phylogenetic tree of selected lamiids. **b** Venn diagram of the number of genes upregulated during interfamily grafting in *Petunia hybrida*, *Nicotiana benthamiana*, and *Ipomoea nil*. Genes were identified using the same criteria as in Fig. 2c. The number of Arabidopsis annotations that best approximated each gene was counted. **c** Expression profiles of genes upregulated in *P. hybrida*, and their homologous genes of *N. benthamiana* and *I. nil* during interfamily grafting. **d** Enriched GO terms related to molecular function in a GO enrichment analysis of the 54 Arabidopsis gene IDs listed in (b). *Ph*, *P. hybrida*, *Nb*; *N. benthamiana*; *In*, *I. nil*.

The interfamily grafting-capable species *P. hybrida* and *N. benthamiana* are both members of Solanaceae, whereas the interfamily grafting-incompetent *I. nil* belongs to a different family in Solanales, Convolvulaceae (Fig. 4a). We hypothesized that the reason for the uneven distribution of interfamily-grafting-capable species in Solanales, despite their close relationships, is that each species has different gene expression regulatory mechanisms for grafting and wound healing. To test this hypothesis, we extracted genes upregulated after graft treatments of the interfamily combinations *N. benthamiana*/Arabidopsis and *I. nil*/Arabidopsis. The gene extraction was conducted in the same manner for the *P. hybrida*/Arabidopsis grafting*.* To adjust the number of extracted genes to comparable levels, the lower limit of expression was set to FPKM >10 for *N. benthamiana* and FPKM >12 for *I. nil*; as a result, 610 and 675 genes were extracted, respectively. We then performed an amino acid homology search of genes of each species against Arabidopsis cDNA reference sequences using TBLASTX and mapped them to the most similar gene. Although the three species are relatively closely related, the percentage of upregulated genes shared between species ranged from 11% to 13%. This low level of overlap demonstrates that each species exerts its own molecular mechanisms upon grafting with an interfamily partner, with only partially identical events (Fig. 4b). Next, we used TBLASTX to identify homologs in *N. benthamiana* and *I. nil* of the 668 upregulated genes of *P. hybrida* interfamily grafting (*N. benthamiana*, 585 genes; *I. nil*, 601 genes), and compared their expression profiles by self-organization maps of their expression (Fig. 4c). In contrast to the 668 genes of *P. hybrida*, their homologous genes in *N. benthamiana* and *I. nil* were not all upregulated; instead, their expression patterns were random. These data further indicate that grafting regulatory mechanisms differed overall among *P. hybrida* and the two other species.

We carried out a gene ontology (GO) enrichment analysis using the TAIR-IDs of the 54 genes in Arabidopsis (Fig. 4d, Supplementary Tables S7–9). A total of 4, 2, and 10 significantly enriched GO terms were respectively detected in biological process, cellular component, and molecular function categories. Under the molecular function category, GO terms related to glucosyltransferase and glycosyltransferase were especially enriched in upregulated genes.

### Elevated *GH9B3* gene expression associated with the interfamily grafting capability of solanaceous plants

**Figure 5:**
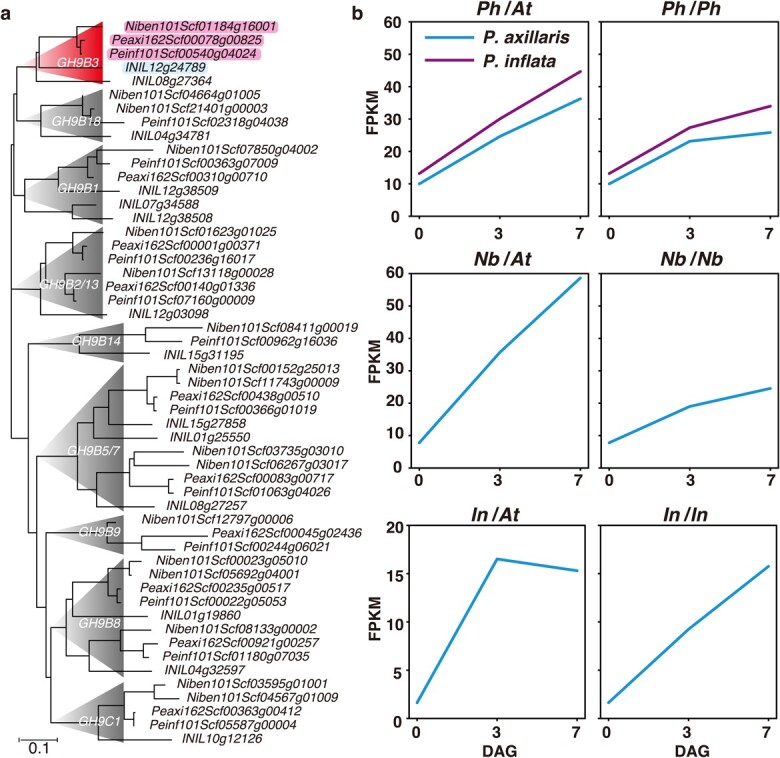
Association of elevated *GH9B3* gene expression with the interfamily grafting capabilities of Solanaceae **a** Phylogeny of *glycosyl hydrolase 9B* genes of *Petunia hybrida*, *Nicotiana benthamiana,* and *Ipomoea nil*. Genes with upregulated expression according to Fig. 4b are highlighted in pink. **b** Expression profiles of *P. hybrida Peaxi162Scf00078g00825* and *Peinf101Scf00540g04024* and *N. benthamiana Niben101Scf01184g16001* genes (all highlighted in a) and *INIL12g24789,* the best approximation of *GH9B3* genes in *I. nil* (highlighted in blue in a), during grafting of these three species onto Arabidopsis (left panels) and self-grafting (right panels). *Ph*, *P. hybrida*; *At*, Arabidopsis; *Nb*, *N. benthamiana*; *In*, *I. nil*.

In the molecular function category, genes associated with the enriched GO term “hydrolase activity, hydrolyzing O-glycosyl compounds” included *AT1G22880* (Supplementary Table S7). Arabidopsis *AT1G22880* encodes the glycosyl hydrolase 9B3 (GH9B3), a member of the β-1,4-glucanase subclade. GH9B3 is upregulated during the grafting of *N. benthamiana* and has been shown to be essential for interfamily grafting of this species; the enzyme is also upregulated during the grafting of *P. japonicum* and is crucial to the parasitization of interfamily host plants [14, 18]. In the phylogenetic tree shown in Fig. 5a, *Peaxi162Scf00078g00825* and *Peinf101Scf00540g04024* of *P. hybrida* are the two GH9B family genes most closely related to the *N. benthamiana GH9B3* gene *Niben101Scf01184g16001* and are considered to be homologs of *GH9B3* in *Petunia* (Fig. 5a). During interfamily grafting, the expression profiles of these two *GH9B3* homologs were similar to that of the *N. benthamiana GH9B3* gene, whose expression continued to rise through 7 DAG. In contrast, the expression of the closest homolog of *I. nil* in the phylogenetic tree, *INIL12g24789*, tended to peak at 3 DAG and then decrease in the interfamily grafting treatment (Fig. 5b). In the case of self-grafting, increased expression of *GH9B3* was maintained up to 7 DAG in all three plant species (Fig. 5b). These results confirm that the upregulation of *GH9B3* is important for the establishment of self-grafting as well as interfamily grafting.

**Figure 6:**
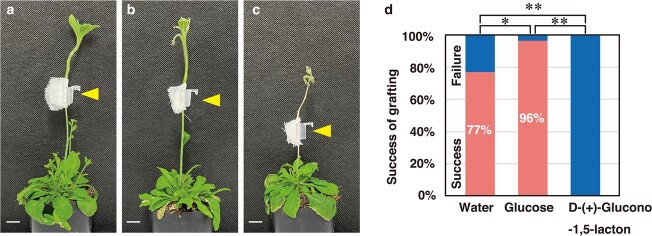
Inhibition of β-1,4-glucanase activity resulting in loss of interfamily grafting capability in *Petunia hybrida***a**–**c** Grafts of *P. hybrida* as a scion on stems of Arabidopsis at 14 DAG in the presence of water as a mock treatment (a), 200 mM glucose as a control (b), and 200 mM D-(+)-glucono-1,5-lactone as an inhibitor of β-1,4-glucanase (c). Arrowheads indicate grafting points. Scale bars, 1 cm. **d** Effect of D-glucono-1,5-lactone or glucose treatment on graft establishment. Differences between sample groups were assessed by pairwise Fisher’s exact tests. ^*^, *P* < 0.05; ^**^, *P* < 0.01 (*n* = 28–35).

To determine the effect of *GH9B3* in *P. hybrida* on graft establishment, the β-1,4-glucanase inhibitor D-glucono-1,5-lactone was applied to the grafting sites of *P. hybrida* and Arabidopsis (Fig. 6). Gluconolactone, a typical lactone in which the hydroxy group at position 1 of glucose has been replaced by a ketone, is a specific inhibitor of β-glucosidase [24]. We obtained a grafting success rate of 96% (27/28) when glucose, which does not inhibit β-glucosidase activity, was applied at a concentration of 200 mM to the grafting sites; this rate was comparable, or even better, than the success rate obtained using water alone as a control, 77% (27/35) (Fig. 6a, b, d). In contrast, D-glucono-1,5-lactone treatment at a concentration of 200 mM resulted in loss of grafting capability (0/31) (Fig. 6c, d).

## Discussion

Two closely related genera in Solanaceae, *Petunia* and *Nicotiana*, have interfamily grafting compatibilities with a wide range of angiosperms [14] (Fig. 1, Table 1). Both genera include flowering ornamentals that are grown in containers and gardens worldwide. Varieties of *P. hybrida* are especially popular because they possess traits such as continuous flowering, amenability to propagation, and disease resistance that simplify their cultivation [25]. In previous study, we found no evidence of any interfamily grafting capability in *I. nil*, a flowering plant in Convolvulaceae, the family in Solanales most closely related to Solanaceae. This result suggests that tissue adhesion with distantly related plant partners can only be facilitated in a subset of plant groups. Our comparison of grafting of these three flowering plant species revealed that each species triggered its own transcriptomic reaction after graft treatments with only some phenomena in common (Fig. 4). This observation is a reminder that each plant group evolves independently and acquires different characteristics. Physiological- and molecular-based classification of commonalities and differences among species therefore allows us to describe individual biological events—for example, interfamily grafting in this study. Similar scenario was also true for the study of parasitism [18]. The plant family Orobanchaceae includes both parasitic and non-parasitic species. Comparison of these two groups has revealed a common feature of parasitic plants when adjoining their tissues to their host plants, which are mostly phylogenetically distant [26]. The tissue-joining capability of the Orobanchaceae root parasite *P. japonicum* with distant plant species can facilitate stem grafting [18]. Given that the evolutionary acquisition of parasitic capacity has occurred 12 times independently in plants [15], the repeated acquisition of tissue-joining capability in plants seems possible.

In previous molecular analyses, wound response was considered to be important for graft establishment [20, 21]. In our GO analysis of 688 genes upregulated in interfamily grafting of *P. hybrida*, the GO terms “meristem structural organization” and “cell division” were enriched only in class-3 genes, which included genes involved in wounding, an event that occurs in grafting. One such gene is *AT1G13870*, the ortholog of the yeast *KTI12* gene encoding a protein named DEFORMED ROOT AND LEAVES1 (DRL1). *drl1* mutant plants exhibit abnormal meristem, root, and leaf development. DRL1 is proposed to be an elongator complex-associated protein, a regulator of cell proliferation, which indicates that this factor may also function in cell proliferation to restore the wounding site [27, 28]. Another enriched GO category included *AT4G17500*, which encodes for ERF-1, a member of the AP2/ERF transcription factor family. The AP2/ERF transcription factor WIND1 is similarly upregulated at wound sites and controls cell dedifferentiation with wounding repair [29]. ERF-1 also regulates root cambium proliferation and stress response in radish [30]. During grafting and wounding of *P. hybrida*, ERF-1 may also have a role in balancing cell proliferation and stress sensitivity in the cambium. In contrast, classes 1 and 2 may include genes related to tissue reunion events other than wound response without tissue reunion (see Materials and methods). *AT1G21270*, which belongs to class 1, encodes a wall-associated kinase 2 (WAK2) protein. WAKs are proteins with an extracellular domain that can bind to cell wall pectin and a serine/threonine kinase domain in the cytoplasm that penetrates the cell membrane and may function in cell wall sensing for the maintenance of cell osmotic pressure [31]. WAK2 may act as a sensor for tissue connection during grafting. *AT3G25070* encodes a RPM1-interacting protein 4 (RIN4). RIN4 has been studied as an immunomodulator in Arabidopsis and is involved in the recognition of external microbial characteristics and bacterial effectors inside plant cells [32]. RIN4 is an intrinsically disordered protein (IDP). Because IDPs can bind to multiple proteins, RIN4 is thought to function as an immune-signaling hub and thus may be involved in self-awareness in grafting. In wounding response, a group of genes related to cell proliferation is actively expressed, whereas the expression of genes related to sensing seems to be biased towards grafting.

In this study, we found genes critical for interfamily grafting by comparing the grafting time-series transcriptomes of three closely related plants belonging to Solanales: *P. hybrida* and *N. benthamiana*, which have interfamily grafting capabilities, and *I. nil*, which does not. In our previous study, we identified specific genes for interfamily grafting that were upregulated in *N. benthamiana* but not in *Glycine max*, which lacks the capability for interfamily grafting. In the present study, we identified genes commonly upregulated in *P. hybrida* and *N. benthamiana* but not in *I. nil* during interfamily grafting. We expected that this identification would narrow the focus to genes more specific to interfamily grafting. GO enrichment analysis of the extracted genes revealed the terms “glucosyltransferase” and “glycosyltransferase” related to flavonoid biosynthesis and metabolism (Supplementary Tables S7 and S9). Many plants in Solanaceae produce and accumulate alkaloid secondary metabolites. Although flavonoids are a different type of secondary metabolite, the GO enrichment of flavonoid genes may reflect Solanaceae family characteristics. In addition, several enriched terms in the molecular function category were involved in flavonoid metabolism (Supplementary Table S9). Flavonoids such as quercetin have antioxidant and anti-inflammatory effects [33, 34]; these effects may thus be involved in the healing of grafting.

*GH9B3*, a graft-associated gene previously discovered in *N. benthamiana* [14, 35, 36, 37], was identified among the 54 annotated genes commonly upregulated in *P. hybrida* and *N. benthamiana*. This discovery is the third reported instance of the *GH9B3* gene being upregulated during interfamily grafting, as its upregulation has been previously reported in *N. benthamiana* and *P. japonicum*. *GH9 β-1,4-glucanase* genes constitute a highly overlapping family, with *GH9B* further divided into 18 additional subclades [36, 38]. In Arabidopsis, sequences of the genes *AT1G71380* (*cel3*) and *AT1G22880* (*cel5*), both members of the *GH9B3* clade, are almost identical [39]. In *P. japonicum*, *GH9B3* clade genes have been further duplicated, with five copies present in that species [18]. In contrast, only one copy has been identified in *N. benthamiana*, whereas two copies have been identified in *P. hybrida*: one from *P. axillaris*, and the other from *P. inflata* (Fig. 5a). Four copies of the *GH9B3* gene are also present in the genome of another parasitic plant in Orobanchaceae, *Striga hermonthica* [18, 40], which suggests that multiple duplication events have occurred in such plants during their evolutionary transition to parasitism. Considering the fact that *GH9B3* is upregulated during self-grafting and may function in the establishment of grafting and given that the upregulation of *GH9B3* is not sustained during interfamily grafting in plants lacking interfamily grafting capability, the upstream regulation of *GH9B3* in *N. benthamiana* and *P. hybrida* of Solanaceae may be different from that in other plant families. Analysis of the upstream factors that regulate *GH9B3* expression is therefore a future challenge.

In this study, we found that D-glucono-1,5-lactone, a specific inhibitor of β-1,4-glucanase, significantly inhibited graft establishment between *P. hybrida* and Arabidopsis. Taking into account that β-1,4-glucanase also contributes to the establishment of parasitism in parasitic plants, D-glucono-1,5-lactone may also inhibit parasitism, a hypothesis that requires future validation. D-glucono-1,5-lactone is not a substrate of β-1,4-glucanase, as its molecular structure is different from that of glucose, a substrate of β-1,4-glucanase. An excess of D-glucono-1,5-lactone should thus inhibit the enzymatic activity of β-1,4-glucanase. As a control experiment for D-glucono-1,5-lactone, we treated grafted plants with glucose. According to our results, glucose may significantly increase the success rate of interfamily grafting. This effect may simply be due to the increased bioactivity of the scion caused by the supply of glucose as a nutrient source. Another possibility is that glucose may support the establishment of grafting through β-1,4-glucanase or by activating additional mechanisms.

On the basis of a series of observations, namely, (1) tissue adhesion between plants from different families has been found in nature, (2) the characters are applicable to the grafting technique, and (3) interfamily-grafting-capable plants share a common molecular basis in that β-1,4-glucanases encoded by *GH9B3* facilitate cell–cell adhesion, we have named this cross-species plant-to-plant grafting technique as “interfamily Partner Accepting Graft” (iPAG) to distinguish from conventional observations that interfamily grafting has mostly been unsuccessful. The iPAG capability of *Petunia* not only expands the cultivation possibilities of this widely popular species itself, but also permits the addition of various traits of *Petunia*, such as its high propagation capacity and environmental stress tolerance, to other flowering plants. In addition, the application of iPAG may facilitate techniques that have not been possible using conventional breeding, such as modifying the flowering season of the grafting partner through the action of a systemic signal florigen. Finally, iPAG, a new category of grafting techniques, will enhance future studies on the mechanisms of grafting.

## Materials and methods

### Plant materials

*P. hybrida* plants were obtained through vegetative propagation. Seeds of *N. benthamiana* were surface sterilized with 5% (w/v) bleach for 5 min, washed three times with sterile water, incubated at 4°C for 3 days, and sown on half-strength Murashige and Skoog medium supplemented with 0.5% (m/v) sucrose and 1% agar. The pH was adjusted to pH 5.8 with 1 M KOH. *I. nil* line Q79 and Arabidopsis ecotype Columbia (Col–0) seeds were directly surface-sown on soil. Arabidopsis and *N. benthamiana* seedlings were grown at 22°C and 27°C, respectively, under continuous illumination of 100 μmol m^−2^ s^−1^.

### Grafting

Stem grafting was performed as described previously [14, 18]. Briefly, wedge grafting was performed on stems, and the grafted plants were initially grown in an incubator at 27°C under continuous light (ca. 30 μmol m^−2^ s^−1^) for a week and then transferred to a plant growth room at 22°C under continuous light conditions (ca. 80 μmol m^−2^ s^−1^). All other plant materials used for stem grafting in this study are listed in Table 1. To apply D-glucono-1,5-lactone or glucose, 200 μL of each chemical solution at a concentration of 200 mM was soaked into a small piece of gauze, wrapped around the grafting site, and fixed with a commercial agricultural grafting clip. No paraffin film was used for wrapping.

### Transcriptome analysis

The grafted or intact plants were harvested at 3 and 7 days after grafting (DAG). Approximately 10 mm–15 mm of graft junction or intact stem tissue at a similar location was sampled. Each biological replicate comprised the pooled tissues from 10 grafts or 10 intact plants. Total RNA was extracted from the samples using a RNeasy Mini kit (Qiagen, Hilden, Germany) following the manufacturer’s protocol. cDNA libraries were prepared with an Illumina TruSeq Stranded Total RNA kit with Ribo-Zero Plant and subjected to 86-bp single-end sequencing on an Illumina NextSeq 500 platform (Illumina, San Diego, CA, USA). Data preprocessing was performed as follows. Raw sequence quality was assessed with FastQC v0.11.4 (http://www.bioinformatics.babraham.ac.uk/projects/fastqc/). Adapters were removed and data were trimmed for quality using Trimmomatic v0.36 with the settings TruSeq3-PE-2.fa: 2:40:15, SLIDINGWINDOW: 4:15, LEADING: 20, TRAILING: 20, and MINLEN: 30 [41]. FastQC quality control was repeated to ensure no technical artifacts were introduced. Trimmed reads were mapped onto the genome assembly using HISAT2 v2.1.0 [42]. The generated SAM files were converted to BAM format and merged using SAMtools v1.4.1 [43]. Gene expression levels (fragments per kilobase of transcript per million fragments mapped, FPKM) were estimated using Cufflinks v2.1.1 with the -G option [44]. Expression fluctuation profiles were generated using Cuffdiff v2.1.1. The reference sequences and version used for mapping and annotation were as follows: *P. axillaris*, *Petunia axillaris* draft genome sequence v1.6.2 [7]; *P. inflata*, *Petunia inflata* draft genome sequence v1.0.1 [7]; *N. benthamiana*, *Nicotiana benthamiana* draft genome sequence v1.0.1 [45]; *A. thaliana*, https://www.arabidopsis.org, TAIR10 genome release; and *I. nil*, http://viewer.shigen.info/asagao/, Asagao_1.2. A GO enrichment analysis was performed with DAVID [46, 47] using Arabidopsis gene IDs. Transcriptome data of grafting were used in a PCA to compare the differences between samples. Python v3.7.4 and its library modules, including NumPy (1.17.2), Pandas (0.25.1), SciPy (1.3.1), Matplotlib (3.1.1), seaborn (0.9.0), and scikit-learn (0.21.3), were used for the PCA and hierarchical cluster classification. The calculation of SOM values was performed with R (3.3.3).

## Acknowledgments

We thank Dr. Eiji Nitasaka and the Morning glory stock center of Kyushu University with support in part by the National Bio-Resource Project of the AMED, Japan for providing *I. nil* Q79 seeds. We thank A. Ishiwata for technical assistance. This work was supported by grants to M.No from the Japan Society for the Promotion of Science Grants-in-Aid for Scientific Research (18H03950, 20H03273, 21H00368, and 21H05657), and the research program on development of innovative technology grants from the Project of the Bio-oriented Technology Research Advancement Institution (BRAIN 28001A and 28001AB).

## Author Contributions

K.K., M.Ni., and M.No. conceived the research and designed the experiments. C.H., K.O., and M.No. performed the grafting experiments and morphological analysis. Y.I. and T.S. performed the RNA-Seq analysis with the support of K.S and T.H. K.K. analyzed the transcriptome data. K.K. and M.No. wrote the manuscript.

## Data availability statement

RNA-seq data are available from the DNA Data Bank of Japan (www.ddbj.nig.ac.jp/) under accession number DRA009936 for *N. benthamiana* and *I. nil*, and from the National Center for Biotechnology Information (https://www.ncbi.nlm.nih.gov) under BioProject ID PRJNA765124 for *P. hybrida*. All other data are available in the main text or the supplementary materials.

## Conflict of interest

Nagoya University has filed for patents regarding the following topics: “Interfamily grafting technique using Petunia,” inventor M.No. (patent publication nos. WO 2016/06018 and JP 2014–212 889); “Grafting facilitation technique using cellulase,” inventors M.No. and K.K. (patent application nos. JP 2019–052727 and JP 2020–042379).

## Supplementary data

Supplementary data is available at *Horticulture Research Journal* online.

## Supplementary Material

Web_Material_uhab056
